# Modeling the Effect of Stress Ratio, Loading Frequency and Fiber Orientation on the Fatigue Response of Composite Materials

**DOI:** 10.3390/polym14142772

**Published:** 2022-07-06

**Authors:** Huidong Ma, Xuezong Bai, Yawei Ran, Xubing Wei, Zongwen An

**Affiliations:** School of Mechanical and Electrical Engineering, Lanzhou University of Technology, Lanzhou 730050, China; mahuidonglut@163.com (H.M.); 15101256903@163.com (X.B.); ranyawei617@163.com (Y.R.); 17361561137@163.com (X.W.)

**Keywords:** composites, fatigue life model, stress ratio, loading frequency, fiber orientation

## Abstract

Fatigue life models are widely used to predict the fatigue behavior at arbitrary cycle counts of composite structures subjected to cyclic or highly dynamic loads. However, their predictive capacity and determination of model parameters are strongly dependent on loading conditions and large experimental efforts. This research aims to develop a new model which uses a single model parameter to predict the variation trend and distribution pattern of fatigue experimental data points subjected to different stress ratios, loading frequencies and fiber orientations. Validation of the model with several sets of experimental data shows that the proposed model is capable of adequately considering the effects of stress ratio, loading frequency and fiber orientation on the fatigue behavior of composite materials and correctly predicting the variation trend of the experimental data points using only one set of model parameters regardless of stress ratios, loading frequencies and fiber orientations.

## 1. Introduction

Due to the superior mechanical properties of fiber-reinforced composite materials, such as low specific gravity, high specific strength (strength-to-weight ratio) and specific stiffness (stiffness-to-weight ratio), their acceptance and applications in wind energy and many other industrial fields are growing year by year [1]. Therefore, understanding the characteristics of these composite materials under realistic service conditions is an essential part of designing efficient and reliable products. However, as the main load-bearing parts of structures made from composite materials experience a wide variety of cyclic or highly dynamic loading conditions, a fatigue phenomenon pertaining to durability and reliability of these anisotropic and heterogeneous composite structures has become a bottleneck limiting their widespread application. Fatigue behavior of composite materials has been recognized as a major focus of research in this field and a considerable number of studies have been reported [2,3,4,5,6,7,8,9,10,11,12].

One way of dealing with the fatigue behavior of composite materials is to develop a mathematical or analytical model capable of predicting the fatigue life of different composite structures under various loading conditions by conducting extensive fatigue experiments [13]. According to Sendeckyj’s recommendations [14], the large number of fatigue models in the current literature can be divided into three categories: fatigue life models [15,16], macroscopic phenomenological models [8,17] and progressive damage models [18]. Hashing and Rotem [15] proposed a fatigue criterion for determining the failure of fibers and matrices based on a static strength failure criterion, where the ultimate strength is a function of the stress level, stress ratio and number of cycles instead of a constant. The expression for the ultimate strength must be obtained from the *S*-*N* curves of various unidirectional laminates under the corresponding load conditions, which means that the criterion is only applicable to single-ply structures under the corresponding load conditions. Moreover, a semi-logarithmic linear relationship between stress levels and number of cycles was proposed by Fawaz and Ellyin [19] and has been commonly accepted. In addition to employing the *S*-*N* curve as a fatigue life model for composite materials, constant life diagrams (CLDs) are also extensively used to predict the fatigue life of composite structures. Similar to CLDs for metallic materials, a linear CLD model was proposed. Although this model is simple and cost-effective, there is a relatively large error between the prediction results and the experimental data [5]. In view of this, many researchers have continued to improve the linear CLD model and derived new models such as the segmented linear model [20] and the segmented non-linear model [16].

Some researchers have also described the damage state of composite structures by monitoring the degradation of their macroscopic properties, from which two prevailing phenomenological models including the residual strength model [8] and the residual stiffness model [17] have been developed. Numerous experimental studies have shown that the residual strength of composite structures decreases slowly at the beginning and then rapidly approaches failure, displaying the so-called “sudden death phenomenon” [8]. Contrary to the degradation of the residual strength, the degradation process of the residual stiffness of composite materials is characterized by a three-stage “fast-slow-fast” [21]. In addition, some researchers have observed degradation processes of other macroscopic material property parameters such as Poisson’s ratio [22], a transverse modulus [23] and an in-plane shear modulus [24]. Further research into how the degradation characteristics of these material property parameters can be considered in phenomenological models is still required.

The progressive damage model is the third fatigue model commonly available in the literature. Combining operations of stress analysis, failure analysis and material property degradation, Shokrieh and Lessard [25] presented a fatigue model applicable to unidirectional carbon fiber-reinforced composites, which is one of the most popular progressive damage models. In recent years, synergistic damage mechanics models, represented by the fatigue model based on continuum damage mechanics proposed by Talreja et al. [18], have received increasing attention due to their clear physical significance and close relevance to the damage mechanism of materials. Although progressive damage models are preferred by researchers for their clear physical definition and wide applicability, their high model complexity and strong dependence on experimental data restrict their engineering application.

Despite considerable efforts that have been made during the last few decades, the diversity of composite constituent systems, the complexity of loading conditions and the coupling of multiple damage modes in composite structures has caused there to still be no commonly accepted fatigue life prediction method that meets the requirements stipulated by Sendeckyj [14]. As reported by Stojković et al. [26], “Most of the models proposed in the literature considered specific stress state, stress ratio and layup, which was the main setback for their further implementation”. The lack of generality of the fatigue life models developed for specific load conditions in most of the literature limits their further application. In other words, existing fatigue life models are overly dependent on loading conditions such as stress levels, stress ratios and loading frequencies and on layup configurations including material type, layup sequence and fiber angle. Just as Kaminski et al. [27] reported, “research efforts should be continued to meet the challenge of developing models with a more generalized applicability in terms of loading conditions and of material selection”.

Along this line, Shokrieh and Lessard [28] pioneered the development of a generalized material property degradation model capable of predicting the fatigue behavior of unidirectional laminates under arbitrary stress ratios and multiaxial stress states by coupling a normalized fatigue life model with a normalized residual strength model. Note that the material properties in this model refer to strength and stiffness. Ma et al. [29] also proposed a new generalized model capable of predicting the material property degradation of composite materials at multiple stress levels using a single set of parameters. This model takes into account the effect of stress level and correctly describes the gradual degradation of strength and stiffness at arbitrary stress levels. Moreover, Kawai [30] developed a general phenomenological model that considers the fiber orientation as well as stress ratio effect on the fatigue behavior of unidirectional composites by introducing the modified non-dimensional effective stress. This model can adequately describe the trend and distribution of fatigue experimental data for unidirectional carbon/epoxy and glass/epoxy composites under cyclic loading with a non-negative stress ratio. The above-mentioned generic models have undoubtedly made a significant contribution to reducing the dependence of fatigue models on loading conditions and layup configurations, but they still take too few influences into account at the same time, and further evolution of generic models is urgent.

Thus, to further reduce the dependence of fatigue life models on loading conditions and layup configurations and to evaluate the fatigue life of fiber-reinforced composite laminates, a model to predict the fatigue behavior of plies at arbitrary stress ratios, fiber angles and loading frequencies is extremely necessary. The present study focuses on the construction of a new fatigue life model that takes into account the effects of stress ratio, fiber orientation and loading frequency. One major difficulty in developing such a model is to incorporate both the loading factors of stress ratio and loading frequency and the layup configuration factor of fiber orientation into the same model formulation. The proposed model follows the fundamental principle that the new model is able to produce different fatigue life curves for varying stress ratios, loading frequencies and fiber orientations to describe the trends and distribution of experimental data reported in the literature.

The present paper is organized as follows: Section 2 summarizes the basic information on all the experimental data used for model validation in the current study. Section 3 analyses the impact of stress ratio, loading frequency and fiber orientation on the fatigue behavior of composite laminates. Section 4 details the process of constructing a new fatigue life model independent of the influencing factors such as stress ratio, loading frequency and fiber orientation. Section 5 verifies whether the proposed model can correctly characterize the fatigue behavior of composite materials regardless of the stress ratio, loading frequency and fiber orientation. Finally, the last section provides a summary of findings and concludes the paper.

## 2. Effect of Stress Ratio, Fiber Orientation and Loading Frequency on Fatigue Life of Composite Laminate

In order to determine the effect of stress ratio, fiber orientation and loading frequency on the fatigue life of composites, the power law expression proposed by Basquin, as shown in Equation (1), is used here to fit the fatigue experimental data.

(1) $$\sigma^{m}N=c$$

where *σ* and *N* refer to applied stress and fatigue life, respectively. *m* and *c* are parameters related to material, stress ratio and loading method. Taking the logarithm of both sides of Equation (1), it can be transformed into:

(2) lgσ=p+qlgN

where *p* and *q* can be determined by Equation (3):

(3) $$p=\frac{\mathrm{lg}c}{m},q=-\frac{1}{m}$$

### 2.1. Effect of Stress Ratio

Andersen et al. [31] performed fatigue tests with a constant load amplitude on load-controlled servo-hydraulic testing machines (Instron 1342 and 1343) and provided a series of experimental data on the mechanical properties of glass fiber-reinforced composite laminates with various layup configurations. Table 1 lists the basic experimental conditions for the material types, layup configurations and stress ratios used in their fatigue tests. The current experimental data were used to investigate the effect of stress ratio on fatigue life of composite structures for two principal reasons: the diversity of composite layup configurations (including unidirectional plies at [0°]_8_ and [90°]_4_, angle ply at [±45°]_S_, and quasi-isotropic ply at [0°/±45°/0°]_S_) and the comprehensiveness of fatigue types (including tension–tension fatigue, compression–compression fatigue, and tension–compression fatigue).

**Figure 1:**
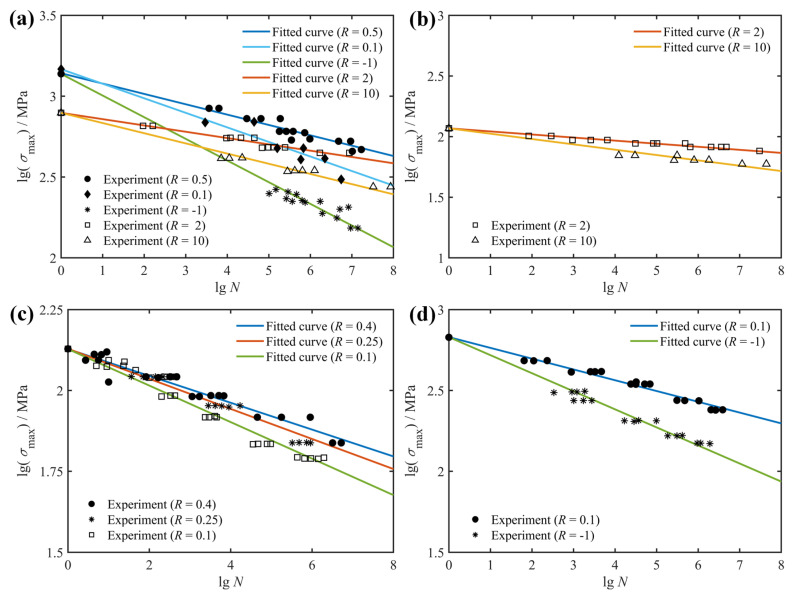
Effect of stress ratio on fatigue life of: (**a**) glass/polyester [0°]_8_; (**b**) glass/polyester [90°]_4_; (**c**) S2/glass/epoxy [±45°]_S_; (**d**) DD5E-glass/epoxy [0°/±45°/0°]_S_, together with experimental data from Ref. [31].

Generally speaking, the fatigue lives and behaviors of composite laminates are dependent on the specific layup configurations as well as realistic service conditions, which are characterized by changes in the fiber angles, stress ratios and loading frequencies. To characterize the effect of stress ratio on the fatigue life of composite laminates, the Basquin model shown in Equation (2) was used in this study to fit the fatigue test data from the literature. Figure 1 depicts the effect of stress ratio on the fatigue life of composite laminates with different layup configurations based on the experimental data of the literature [31]. It is essential to note that when studying the effect of stress ratio on the fatigue response of composite materials, only the stress ratio is variable whereas both the loading frequency and fiber orientation are fixed. As can be seen from Figure 1a, the fatigue life of glass fiber-reinforced composite laminates with a stacking order of [0°]_8_ increases with the increasing stress ratio in the uniaxial tension–tension fatigue test, whereas in the uniaxial compression–compression fatigue test, the fatigue life of unidirectional laminates with a stacking order of [0°]_8_ decreases with an increase in the stress ratio. In addition, the uniaxial tensile fatigue life (*R* = 0.1 and 0.5) of unidirectional laminates of fiber-reinforced composites is longer than the uniaxial compression fatigue life (*R* = 2 and 10), and the uniaxial tension–compression fatigue life (*R* = −1) is the shortest. From the analysis of microscopic damage of composite materials, it can be seen that under uniaxial tensile loading, the failure mode that determines the fatigue life of unidirectional laminates is fiber fracture, whereas under uniaxial compression loading, transverse cracking of the matrix and subsequent delamination damage play a highly important role in the fatigue failure of unidirectional laminates. Hence, the uniaxial tensile fatigue performance of unidirectional laminates is superior to the uniaxial compression fatigue life.

Figure 1b shows the fatigue life of the off-axis laminate with the stacking sequence of [90°]_4_ under compression–compression loading. From the fitted results, the fatigue life of the glass/polyester composite laminate decreases as the stress ratio rises. Since the transverse mechanical properties of fiber-reinforced composites are mainly determined by the matrix and the matrix–fiber interface, the dominant failure modes of [90°]_4_ unidirectional laminates are matrix cracking and interface debonding.

Figure 1c is the fitted fatigue life of the S2/glass/polyester angle laminate subjected to tension–tension dynamic stresses, together with corresponding experimental data. Apparently, similar to unidirectional laminates at [0°]_8_, the fatigue life of angled laminates at [±45°]s is positively related to the stress ratio when they are subjected to tension–tension fatigue loading. Figure 1d compares the fatigue life fitting and prediction results under uniaxial tension–tension loading with a stress ratio of 0.1 and fully reversed loading, and the results show that fully reversed dynamic loading is more damaging to the structure than tension–tension dynamic loading. Together, for these composite structures involved in the fatigue tests in Figure 1, the fatigue life corresponding to tension–tension fatigue loading increases with increasing stress ratio, whereas the fatigue life corresponding to compression–compression fatigue loading decreases with increasing stress ratio, and the fatigue life is the smallest for fully reversed loading.

### 2.2. Effect of Fiber Orientation

To understand the fiber orientation effect on the fatigue life of composites, El Kadi and Ellyin [32] and Kawai [30] conducted dynamic tests using a load-controlled method on glass fiber-reinforced unidirectional laminates with 0°, 19°, 45°, 71° and 90° fiber orientations and carbon fiber-reinforced unidirectional laminates with 0°, 10°, 15°, 30°, 45° and 90° fiber orientations, respectively. The corresponding basic conditions for fatigue testing of unidirectional laminates with different fiber orientations are given in Table 2. Although their fatigue tests also considered the effect of stress ratio on the fatigue life of the composites, the composite layup configuration used in their fatigue tests had only unidirectional laminates, which made it unsuitable for analyzing the effect of the stress ratio but ideal for determining the fiber angular orientation effect of the composites. It must be noted that the parameter used to characterize fatigue life in these two fatigue tests is the number of reversals to failure rather than the number of load cycles, and they are related by the fact that the number of reversals to failure is twice the number of load cycles.

**Figure 2:**
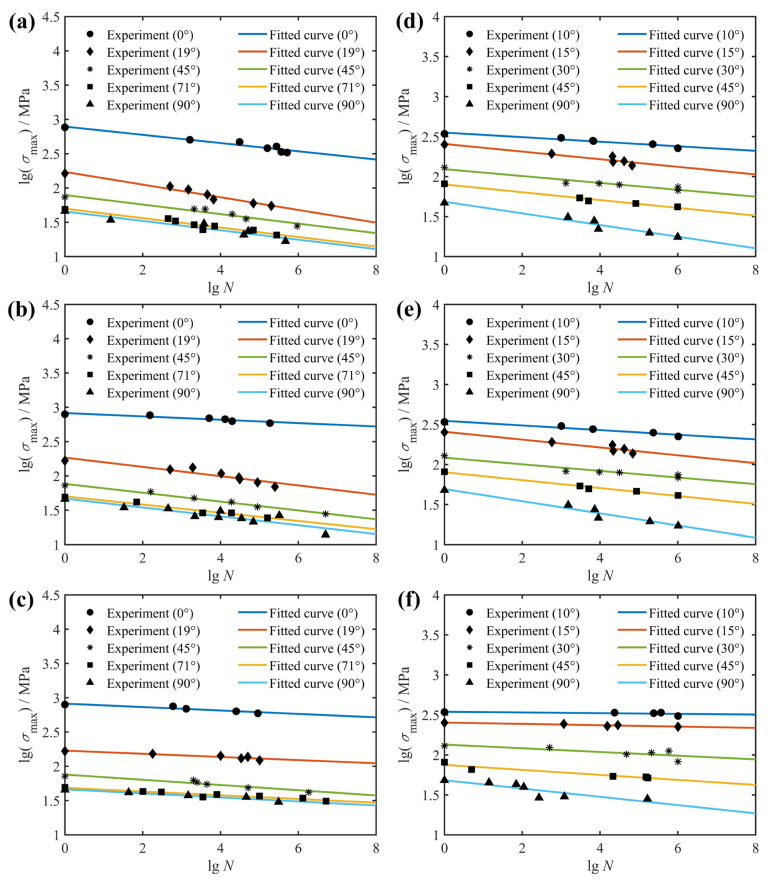
Effect of fiber orientation on fatigue life of unidirectional E-glass/epoxy [32] (**a**–**c**) and T800H/2500 [30] (**d**–**f**) specimens at stress ratio of: (**a**) *R* = −1; (**b**) *R* = 0; (**c**) *R* = 0.5; (**d**) *R* = −1; (**e**) *R* = 0.1; (**f**) *R* = 0.5.

The fatigue behavior of composite laminates also depends on the fiber orientation in terms of the composite layup configuration. Figure 2 presents the off-axis fatigue behavior of the unidirectional E-glass/epoxy composite (*θ* = 0, 19, 45, 71 and 90°; *R* = −1, 0, 0.5) and unidirectional T800H/2500 composite (*θ* = 10, 15, 30, 45 and 90°; *R* = −1, 0.1, 0.5). From the *S*-*N* curves fitted to the experimental data, it is clear that the fatigue life of unidirectional laminates decreases with increasing fiber orientation. This is due to the fact that the failure mode of the unidirectional laminate changes from fiber-dominated failure to matrix-dominated failure as the fiber orientation increases from 0° to 90°, and the mechanical properties of the fiber are significantly superior to those of the matrix.

### 2.3. Effect of Frequency

Andersen et al. [31] also performed fatigue tests at different loading frequencies and provided experimental results on the mechanical properties of glass fiber-reinforced composite laminates with various frequencies as given in Table 3. According to static test standard ASTM D3039 [33] and fatigue test standard ASTM D3479 [34], Justo et al. [35] performed static and fatigue tension tests using the Instron 4482 electromechanical testing machine and Instron 8801 hydraulic testing machine, respectively. The basic experimental information including material type, layup configuration, static ultimate strength and test frequency are listed in Table 3. All dynamic tests are implemented by means of load control with a stress ratio of *R* = 0.1 and sinusoidal variation.

**Figure 3:**
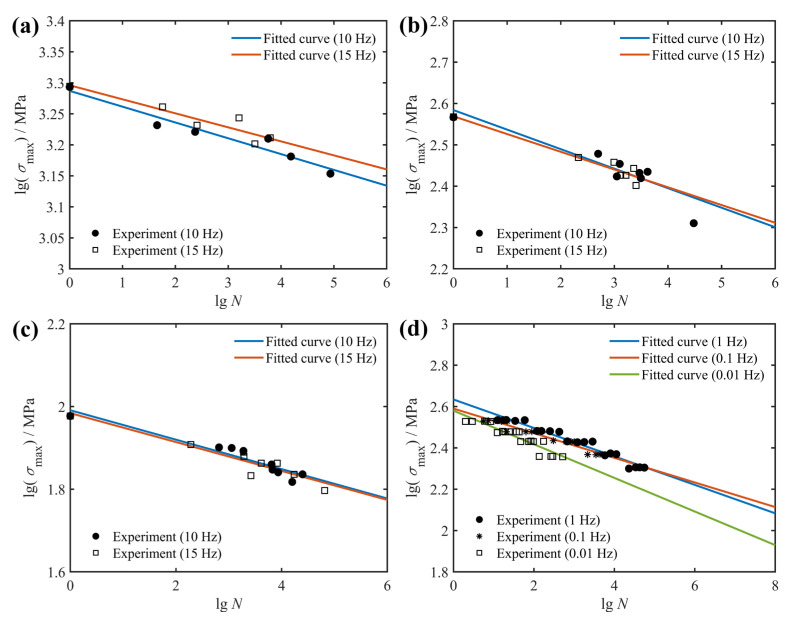
Effect of loading frequency on fatigue life of: (**a**) graphite/epoxy [0°]_4_; (**b**) graphite/epoxy [15°]_4_; (**c**) graphite/epoxy [45°]_4_; (**d**) E-glass/epoxy [0°/90°]_S_, together with experimental data from Ref. [35] (**a**–**c**) and Ref. [31].

To accelerate the process of studying the fatigue behavior of composite structures, most researchers have shortened the fatigue test time by loading at higher frequencies. This raises the challenge that the effect of the loading frequency itself affects the fatigue behavior of composite materials [35,36]. In Figure 3, fitted *S*-*N* curves for composite laminates at various loading frequencies and fatigue experimental data from the literature [31,35] are given. From the results in Figure 3a–c, it is observed that slight variations in the loading frequency have little or negligible effect on the fatigue life of unidirectional laminates with off-axis angles. In contrast, the loading frequency obviously has a significant effect on the fatigue behavior of the composite laminate when the magnitude of the loading frequency change is relatively large (increased by a factor of 10 and 100), as shown in Figure 3d. Additionally, the fatigue life increases with the increasing frequency. Contrary to this rule, high-frequency fatigue tests by some scholars [1] have shown that high loading frequencies (e.g., 20 kHz) lead to a considerable temperature rise, and this temperature rise in turn reduces the fatigue life of the composite materials. Therefore, fully determining the effect of loading frequency on the fatigue behavior of composites is an extremely challenging task.

## 3. Modelling

It is assumed that the residual strength of the composite material decays continuously with load cycles and follows a power law [37]:

(4) $$\frac{d\sigma_{n}}{dn}=-a_{1}n^{-b_{1}}$$

where *σ_n_* is the residual strength of the composite material after *n* cycles; *a*_1_ and *b*_1_ are the positive definite constants.

According to the correlation (*t* = *n*/*f*) between the loading time *t* and the load cycle count *n* at constant frequency loading, Equation (4) can be rewritten as a function of time as follows:

(5) $$\frac{d\sigma }{dt}=-a_{2}t^{b_{2}}$$

where *a*_2_ and *b*_2_ are the positive definite constants. Based on the hypothesis that the temperature of the specimen is controlled below the glass transition temperature of the specific composite material [38], the material constant *a*_2_ is simultaneously influenced by the ultimate tensile strength (*σ_u_*), the stress ratio (*R*) and the stress level (*σ*_max_), as expressed in Equation (6):

(6) $$a_{2}=A\cdot F\left(\sigma_{u},R,\sigma_{\max}\right)$$

where *A* is a material constant that depends on the material properties, loading type, temperature of the specimen and moisture content. *F* (*σ_u_*, *R*, *σ*_max_) is a fatigue failure function with *σ_u_*, *R* and *σ*_max_ as control parameters.

Hertzberg and Manson [39] and Sendeckyj [14] established the formula for the fatigue failure function *F* (*σ_u_*, *R*, *σ*_max_) in Equation (6) under fully reversed loading and tension–tension fatigue loading conditions, as shown in Equation (7):

(7) $$F\left(\sigma_{u},R,\sigma_{\max}\right)=\sigma_{u}{}^{1-\gamma }{\left(1-\psi \right)}^{\gamma }\sigma_{\max}{}^{\gamma }$$

where *γ* and *ψ* are calculated by Equations (8) and (9), respectively. Note that *θ* in Equation (8) represents the minimum angle between the fiber direction of the composite laminate and the loading direction.

(8) γ=1.6−ψsinθ

(9) $$\left\{ \begin{array}{l} \psi =R,R<1\\ \psi =\frac{1}{R},R>1 \end{array}\right.$$

The fatigue life of a composite laminate, defined as the total number of load cycles (*N*) during which the residual strength (*σ*) degrades from static strength (*σ_u_*) to peak stress (*σ*_max_), can be determined by integrating Equation (5) from *t* = *T*_1_ (*n* = 1) to *t* = *T_N_* (*n* = *N*) as given in Equation (10):

(10) $${\left[\sigma \right]}_{\sigma_{u}}^{\sigma_{\max}}=-\frac{a_{2}}{-b_{2}+1}{\left[t^{-b_{2}+1}\right]}_{t=T_{1},n=1}^{t=T_{N},n=N}$$

Substituting Equations (6) and (7) into Equation (10), the following expression can be obtained:

(11) $$\sigma_{\max}-\sigma_{u}=-\frac{A\sigma_{u}^{1-\gamma }\sigma_{\max}^{\gamma }{\left(1-\psi \right)}^{\gamma }}{-b_{2}+1}\frac{1}{f^{-b_{2}+1}}\left(N^{-b_{2}+1}-1\right)$$

For the sake of simplifying expression, the linear combination of the material constants in Equation (11) is replaced as in Equation (12); thus, Equation (11) can be reorganized as Equation (13).

(12) $$\alpha =\frac{A}{-b_{2}+1},\beta =-b_{2}+1$$

(13) $$N={\left[1+\frac{f^{\beta }}{\alpha }\left(\frac{\sigma_{u}}{\sigma_{\max}}-1\right){\left(\frac{\sigma_{u}}{\sigma_{\max}}\right)}^{\gamma -1}\frac{1}{{\left(1-\psi \right)}^{\gamma }}\right]}^{\frac{1}{\beta }}$$

Further reorganization of Equation (13) yields the logarithmic form of the fatigue life as follows:

(14) $$\mathrm{lg}N=\frac{1}{\beta }\mathrm{lg}\left[1+\frac{f^{\beta }}{\alpha }\left(\frac{\sigma_{u}}{\sigma_{\max}}-1\right){\left(\frac{\sigma_{u}}{\sigma_{\max}}\right)}^{\gamma -1}\frac{1}{{\left(1-\psi \right)}^{\gamma }}\right]$$

## 4. Validation Examples

To verify that the model shown in Equation (14) can describe the fatigue behavior of composite laminates regardless of the stress ratio, fiber orientation and loading frequency, examples are given here to address each of these three influencing factors. The basic idea of all the examples is to use a set of model parameters under one loading condition (layup configuration) to predict the fatigue behavior of the composite laminate under the remaining loading conditions (layup configurations).

### 4.1. Fatigue Life Prediction for Variable Stress Ratios

By applying regression analysis, all model parameters considering ratio effect are obtained and listed in Table 4. It should be noted that since the uniaxial tensile fatigue performance of unidirectional laminates with a stacking order of [0°]_8_ is better than the uniaxial compressive fatigue performance, the current study considers that the fatigue performance under fully reversed loading is more influenced by the uniaxial tensile fatigue performance. Hence, the fully reversed fatigue life for the unidirectional laminate of [0°]_8_ is predicted based on the model parameters under uniaxial tensile conditions with a stress ratio of 0.1.

**Figure 4:**
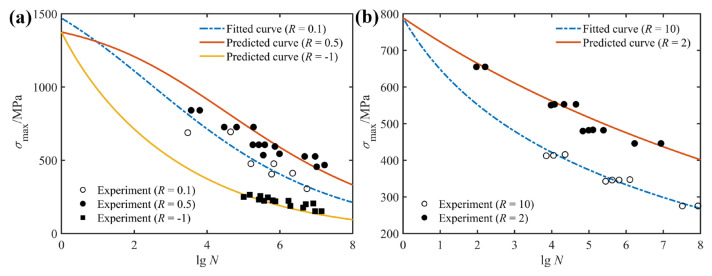
Fitted and predicted *S*-*N* curves of unidirectional glass/polyester [0°]_8_, along with experimental data from Ref. [31]: (**a**) Tension–tension and tension–-compression fatigue; (**b**) compression–compression fatigue.

**Figure 5:**
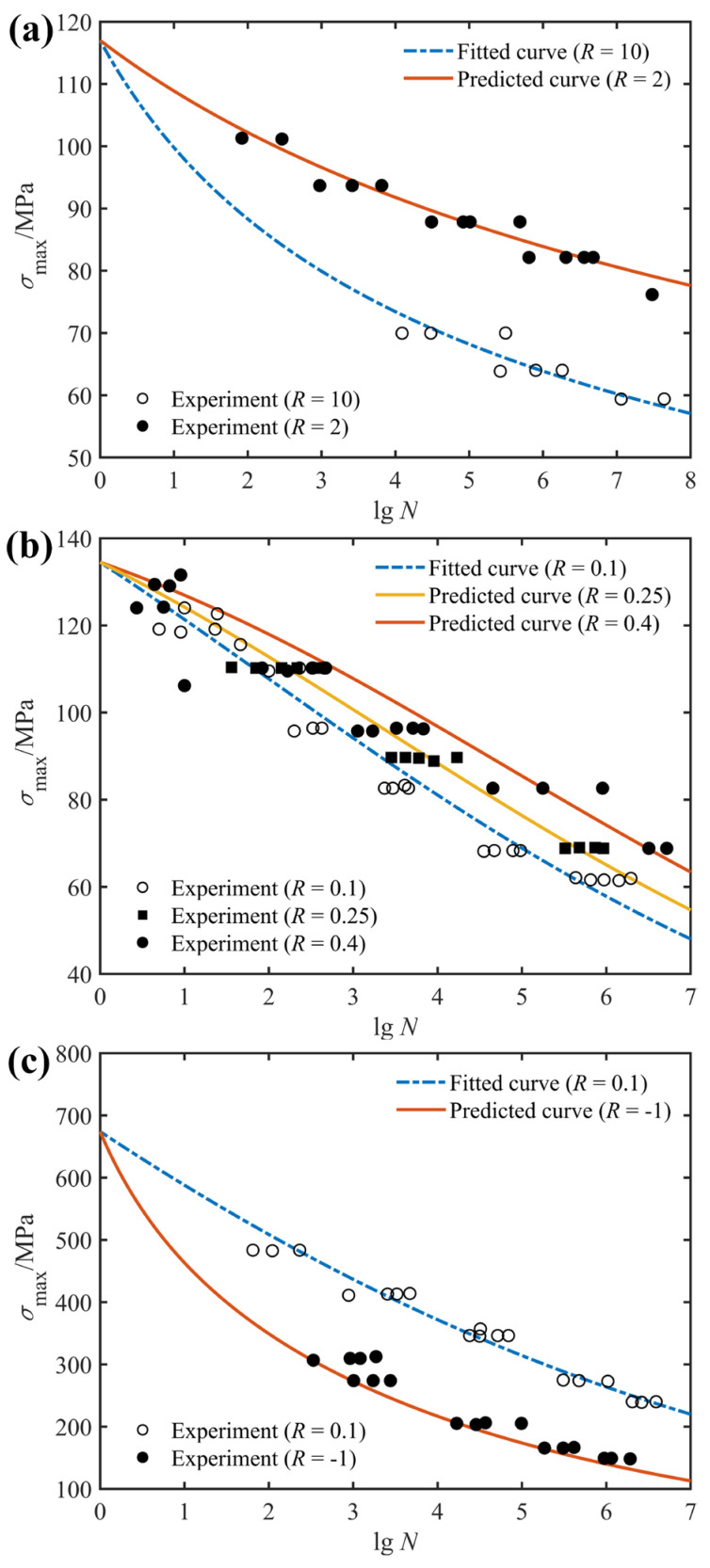
Fitted (*R* = 10) and predicted (*R* = 2) *S*-*N* curves of: (**a**) unidirectional glass/polyester [90°]_4_; (**b**) S2/glass/epoxy with angle-ply configuration of [±45°]_S_; (**c**) DD5E-glass/epoxy with stacking sequence of [0°/±45°/0°]_S_; along with experimental data from Ref. [31].

Comparisons between predicted *S*-*N* curves and experimental data at various stress ratios are presented in Figure 4 and Figure 5. Among these, the blue dotted lines indicate the results of model fitting, which were used to obtain the model parameters, whereas the solid lines denote the predicted results for fatigue life at different stress ratios. For the experimental data, a hollow circle indicates that the set of data is used to fit the model parameters, whereas a solid symbol means that the set of data is used to verify the prediction accuracy of the proposed model. Figure 4 represents the fitted and predicted *S*-*N* curves of unidirectional glass/polyester with a stacking sequence of [0°]_8_, as well as experimental data under tension–tension (Figure 4a), compression–compression (Figure 4b) and tension–compression (Figure 4a) loading. Apparently, all experimental data are closely distributed in the vicinity of the predicted *S*-*N* curves, which suggests that the effect of stress ratio on the fatigue behavior of composite laminates is adequately described by the proposed model with only one set of model parameters. Furthermore, the intercept of the *S*-*N* curve on the vertical axis refers to the static ultimate strength at the corresponding loading condition.

Figure 5a plots fitted and predicted *S*-*N* curves for the unidirectional glass/polyester of [90°]_4_ under the compression–compression loading condition. Comparing the predicted curves with experimental data makes it clear that the proposed model correctly describes the variation trend of the selected data. Similar comparisons between predicted *S*-*N* curves and experimental data for the S2/glass/epoxy with the angle-ply configuration of [±45°]_S_ and the DD5E-glass/epoxy with a stacking sequence of [0°/±45°/0°]_S_ at various stress ratios are given in Figure 5b,c. Taking into account statistical dispersion of the fatigue data, it is also seen that the characteristics of fatigue behavior for the selected composite laminates have been satisfactorily revealed and the effect of the stress ratio on fatigue behavior for the selected composite laminates is adequately described by the proposed model.

### 4.2. Fatigue Life Prediction for Variable Fiber Orientations

In addition to load factors such as stress ratios that directly affect the fatigue behavior of composite laminates, the layup configuration of the composite structure (especially the unidirectional composite laminate) such as fiber orientation also makes an essential contribution to its mechanical properties. The proposed model considers fiber orientation as an influencing factor through the parameter *γ*. It is to be noted that for unidirectional laminates, the fiber orientation is the off-axis angle, whereas for other laminates, the fiber orientation represents the minimum angle between the fiber direction and the loading direction. The model parameters obtained from the regression analysis, which were also used to predict the fatigue behavior of unidirectional laminates with other fiber orientations based on the experimental data available in the literature [30,32], are listed in Table 5.

**Figure 6:**
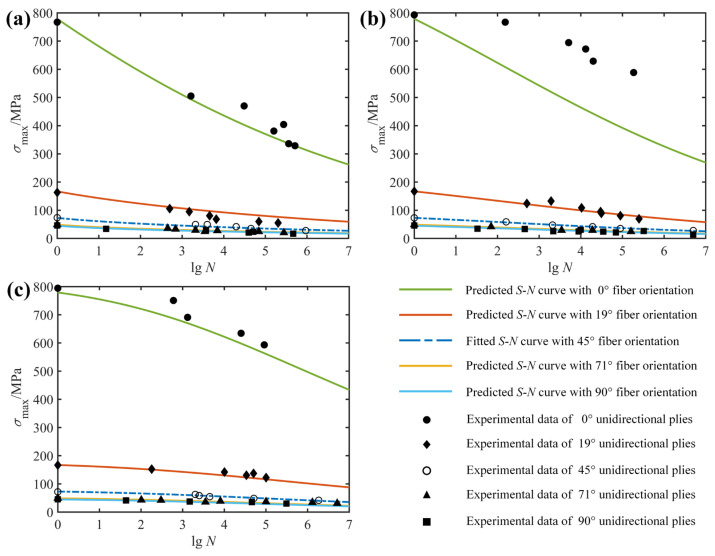
Fitted (45°) and predicted (other angles) *S*-*N* diagrams of unidirectional E-glass/epoxy specimens, together with experimental data from Ref. [32]: (**a**) *R* = −1; (**b**) *R* = 0; (**c**) *R* = 0.5.

**Figure 7:**
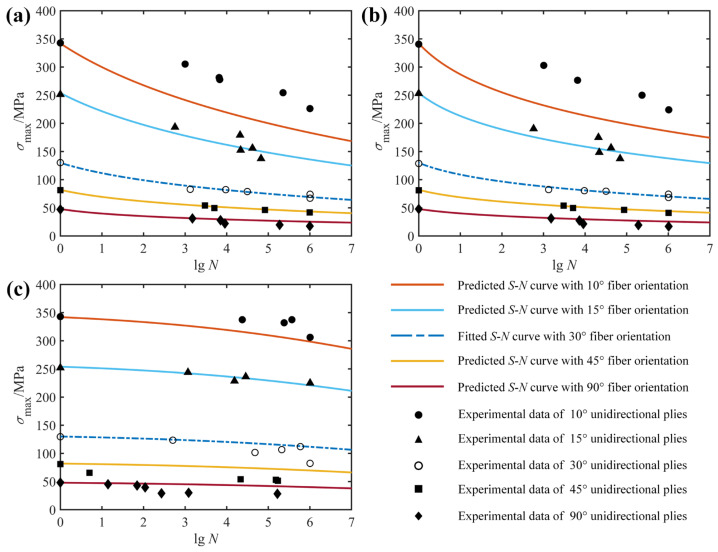
Fitted (30°) and predicted (other angles) *S*-*N* diagrams of unidirectional T800H/2500 specimens, together with experimental data from Ref. [30]: (**a**) *R* = −1; (**b**) *R* = 0.1; (**c**) *R* = 0.5.

In order to verify that the proposed model takes into account the effect of fiber orientation, two examples are given here. The fitted and predicted *S*-*N* curves together with the experimental data for the unidirectional laminates E-glass/epoxy and T800H/2500 with different fiber orientations are shown in Figure 6 and Figure 7, respectively. Similarly, the blue dotted lines indicate the results of the model fitting, which were used to obtain the model parameters, whereas the solid lines denote the predicted results for fatigue life at different fiber orientations. In Figure 6, the fatigue data at the 45° fiber orientation are used to fit and obtain a set of model parameters that are then applied to predict fatigue life curves at other fiber orientations (0°, 19°, 71° and 90°). Although the *S*-*N* prediction curves in both Figure 6a,c correctly describe the trend and distribution of the experimental data, what is striking in this figure is not all of the *S*-*N* curves pass precisely through the middle part of the observed data points, such as the fatigue life curve at fiber orientation of 0° in Figure 6b. Significant discrepancies occur when using *S*-*N* curves fitted to experimental results from weak specimens with relatively low strength to predict the fatigue life of strong specimens with high strength. Fortunately, the predicted fatigue life curve is always on the safe side of the experimental data, just like the linear residual strength model widely used in engineering [40]. Furthermore, the remaining fatigue life curves all reflect the variation trend of the experimental data at the corresponding fiber orientation well. Thus, it can be concluded from Figure 6 that the effect of fiber orientation on the fatigue behavior of unidirectional composite laminates has been adequately considered.

A similar example for the unidirectional T800H/2500 composite at different fiber orientations was conducted and presented in Figure 7. In this example, a set of model parameters obtained by regression analysis according to the fatigue data at the 30° fiber orientation was used to predict fatigue life curves at other fiber orientations (10°, 15°, 45° and 90°). What is clear in Figure 7 is that the predicted *S*-*N* curves for the 10° fiber orientation do not cross exactly in the middle of the experimental data points and always lie on the safe side of the experimental data, just like the predicted *S*-*N* orientation at 0° in Figure 6b. At the same time, the rest of the *S*-*N* curves pass mainly through the middle part of the fatigue data points and, unlike the fatigue life curves at the 10° fiber orientation, never go outside the scatterband of observed fatigue lives. Therefore, the validation of the two examples presented in Figure 6 and Figure 7 indicates that the shortcoming of the currently proposed model is that the predicted and experimental results deviate markedly when the strength of the specimen used to fit the life curve differs greatly from that of the predicted specimen. However, taking into account the conservative design of composite structures and the statistical dispersion of fatigue data, the effect of fiber orientation on the fatigue behavior of composite laminates is considered to be adequately described.

### 4.3. Fatigue Life Prediction for Variable Frequencies

**Figure 8:**
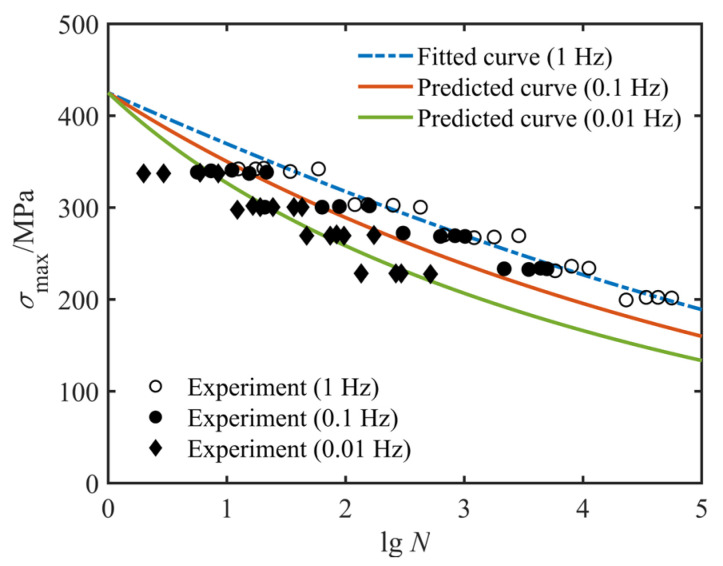
Fitted (1 Hz) and predicted (0.1 Hz and 0.01 Hz) *S*-*N* diagrams of cross-ply E-glass/epoxy specimens based on experimental data from Ref. [31].

**Figure 9:**
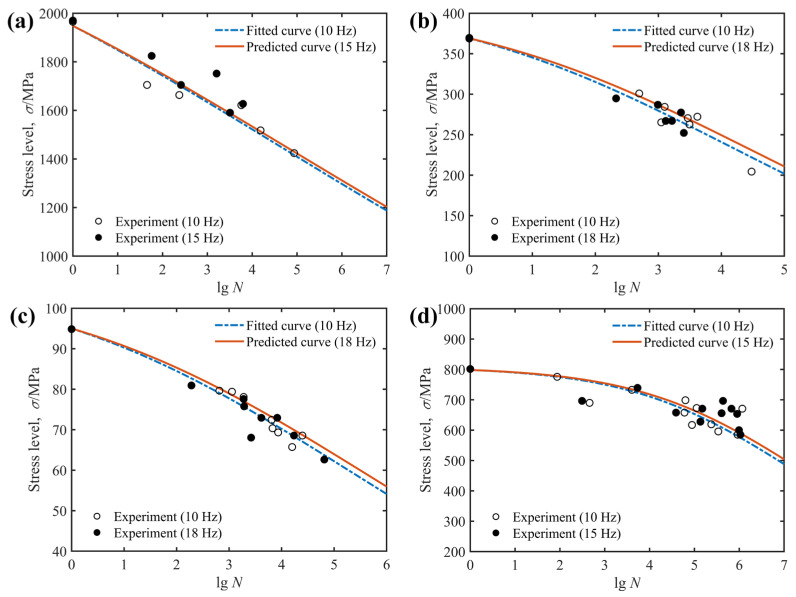
Fitted and predicted *S*-*N* diagrams of graphite/epoxy specimens based on experimental data from Ref. [35]: (**a**) [0°]_4_; (**b**) [15°]_4_; (**c**) [45°]_4_; (**d**) [0°/90°]_3_.

The results shown in Figure 3d indicate that the loading frequency has a significant effect on the fatigue behavior of composite laminates in the case of a considerable variation of loading frequency. Moreover, the loading rate makes a substantial contribution to the stress–strain relationship of the composites. These facts accordingly indicate that the fatigue behavior of composite structures is also governed by the loading frequency. To demonstrate that the proposed model has the ability to take into account the effect of loading frequency, two validation cases shown in Figure 8 and Figure 9 were conducted and the model parameters obtained from the model fitting process are listed in Table 6. Note that in both figures, the blue dotted lines are the *S*-*N* curves obtained to fit the model parameters, and the corresponding experimental data are marked by hollow circles. In contrast, the solid line and the solid symbols indicate the predicted fatigue life curves and the experimental data to verify the correctness of these curves, respectively.

Fitted and predicted fatigue life curves for E-glass/epoxy cross-ply composites at different loading frequencies are plotted in Figure 8. It is shown in this figure that the experimental data at 0.1 Hz and 0.01 Hz loading frequencies are uniformly scattered in the vicinity of the predicted fatigue life curves. Similar conclusions for graphite/epoxy composites with a stacking sequence of [0°]_4_, [15°]_4_, [45°]_4_ and [0°/90°]_3_ can be drawn from Figure 9. Unlike the results in Figure 8, the smaller variation in loading frequency in Figure 9 has a negligible effect on the fatigue life of the composite laminate. However, regardless of whether the variation of loading frequency plays a significant or slight effect on the fatigue behavior of composite laminates, the proposed model correctly characterizes the variation trend of experimental data at different loading frequencies with only one set of model parameters. Consequently, the current study believes that the proposed model can adequately describe the effect of loading frequency on the fatigue behavior of composite laminates.

## 5. Conclusions

This study investigated the effect of stress ratio, loading frequency and fiber orientation on the fatigue behaviors of composite laminates and proposed a new fatigue life model by taking into account these influencing factors. The results obtained can be summarized as follows:

(1) Considering the effect of stress ratio and loading frequency on fatigue life, the proposed model provides an accurate prediction of fatigue life at the remaining stress ratios or loading frequencies based on model parameters determined from experimental data at a specific stress ratio or loading frequency. Taking into account the statistical dispersion of fatigue data, the effect of stress ratio and loading frequency on the fatigue behavior of composite laminates is considered to be adequately described by the proposed model.

(2) The apparent discrepancy between the predicted curves and the experimental data when the proposed model describes the effect of fiber orientation on the fatigue behavior of composites indicates that the model parameters determined for medium strength specimens (i.e., specimens with a fiber orientation of 30° or 45°) are not suitable for predicting the fatigue life of specimens with significantly higher strengths (i.e., specimens with a fiber orientation of 0° or 10°) than these specimens, which is a shortcoming of the current model and an aspect for future improvement.

## Figures and Tables

**Table 1:** Composite layup configuration and ultimate strength from Ref. [31].

Material	Layup Configuration	Stress Ratio *R*	Ultimate Strength /MPa
Glass/Polyester	[0°]_8_	0.1	1470
		0.5	1375
		−1	1375
		10	789
		2	789
Glass/Polyester	[90°]_4_	10	117
		2	117
S2/Glass/Epoxy	[±45°]_S_	0.1	134.5
		0.25	134.5
		0.4	134.5
DD5E-Glass/Epoxy	[0°/±45°/0°]_S_	0.1	674
		−1	674

**Table 2:** Fatigue test conditions for unidirectional laminates with different fiber orientations.

Data Source	Material Type	Loading Frequency/Hz	Stress Ratio *R*	Fiber Orientation/°	Ultimate Strength of each Orientation/MPa
Ref. [32]	E-Glass/Epoxy	3.3	0	0, 19, 45, 71, 90	779, 167, 73, 49, 45
			0.5		
			−1		
Ref. [30]	T800H/2500	10	0.1	10, 15, 30, 45, 90	342, 254, 130, 82, 48
			0.5		
			−1		

**Table 3:** Tensile strength and test frequency of unidirectional and cross plies from Ref. [35].

Data Source	Material Type	Layup Configuration	Tensile Strength /MPa	Frequency /Hz
Ref. [31]	E-Glass/Epoxy	[0°/90°]_S_	425	1
				0.1
				0.01
Ref. [35]	Graphite/Epoxy (AS4/8552)	[0°]_4_	1948	10
				15
		[15°]_4_	369	10
				18
		[45°]_4_	95	10
				18
		[0°/90°]_3_	798.3	10
				15

**Table 4:** Determination of model parameters considering stress ratio effect.

Materials and Layup Configuration	Stress Ratio *R*		Model Parameters	
	Fitting	Prediction	*α*	*β*
Glass/Polyester [0°]_8_	0.1	0.5, −1	0.202	0.256
	10	2	1.631	0.07089
Glass/Polyester [90°]_4_	10	2	125	0.0007594
S2/Glass/Epoxy [±45°]_S_	0.1	0.25, 0.4	0.3033	0.1593
DD5E-Glass/Epoxy [0°/±45°/0°]_S_	0.1	−1	0.446	0.1527

**Table 5:** Determination of model parameters considering fiber orientation effect.

Materials and Layup Configuration	Stress Ratio	Fiber Angle *θ*/°		Model Parameters	
		Fitting	Prediction	*α*	*β*
Unidirectional E-Glass/Epoxy	−1	45	0, 19, 71, 90	0.15	0.1485
	0			0.2896	0.1729
	0.5			0.1879	0.2354
Unidirectional T800H/2500	−1	30	10, 15, 45, 90	0.3263	0.06516
	0.1			2.3	0.06035
	0.5			0.1182	0.1405

**Table 6:** Determination of model parameters considering loading frequency effect.

Materials and Layup Configuration	Loading Frequency *f*/Hz		Model Parameters	
	Fitting	Prediction	*α*	*β*
E-Glass/Epoxy cross-ply	1	0.1, 0.01	0.4146	0.1665
Graphite/Epoxy [0°]_4_	10	15	0.3201	0.1
Graphite/Epoxy [15°]_4_	10	18	0.22	0.2098
Graphite/Epoxy [45°]_4_	10	18	0.2	0.1647
Graphite/Epoxy [0°/90°]_3_	10	15	0.02888	0.2585
